# 

**Published:** 1882-07

**Authors:** 


### CONTENTS:

PAGE.

ORIGINAL COMMUNICATIONS:

I. Treatment of Erysipelas. By G. Frank Lydston, M.D. .... 1
II. On the Causes and Best Means of Preventing the Bowel Affections of Young Children. By N. S. Davis, M.D., Etc. .... 15
III. Valedictory Address to the Graduating Class of the Woman's Medical College. By I. N. Danforth, M.D. .... 21
IV. Death from Thirty Grains of Dover's Powder. By S. Bailey, M.D. .... 26
V. Dr. H. H. Clark's New Suture Button .... 27
VI. Gastric Dyspepsia. By Wm. Henry, M D. .... 29

CLINICAL REPORTS:

VII. A Case of Epileptiform Neuralgia. Reported by E. P. Davis, M.D. .... 32

FOREIGN CORRESPONDENCE:

VIII. Vienna Letter .... 37

DOMESTIC CORRESPONDENCE:

IX. New York Letter .... 40
X. Letter from Elgin .... 45
XI. Letter from Iowa .... 46

PAGE.

SOCIETY REPORTS:

XII. Chicago Medical Society .... 48

ORIGINAL TRANSLATIONS .... 53

EDITORIAL:

Congressional Appropriations for Medical and Health Purposes .... 62
American Medical College Association .... 63

ITEMS .... 47, 54, 64, 111

SELECTIONS:

A Study of Blood. By Lester Curtis, M.D. .... 65
Report of the Post-Mortem Examination of the Body of Charles J. Guiteau .... 73
The Contagious Diseases of Domestic Animals. By E. M. Hunt, M.D. .... 78
Remarks on the Treatment of Gunshot Wounds of the Abdomen in Relation to Modern Peritoneal Surgery. By J. Marion Sims, M.D. .... 95
A Study of the Periods of Incubation of the Communicable Diseases. By B. W. Richardson .... 103

ABSTRACTS .... 108

ANNOUNCEMENTS FOR THE MONTH .... 112

NOTICE TO CORRESPONDENTS .... *advts.*

